# A Novel Phase Portrait for Neuronal Excitability

**DOI:** 10.1371/journal.pone.0041806

**Published:** 2012-08-08

**Authors:** Guillaume Drion, Alessio Franci, Vincent Seutin, Rodolphe Sepulchre

**Affiliations:** 1 Neurophysiology Unit and GIGA Neurosciences, University of Liège, Liège, Belgium; 2 Department of Electrical Engineering and Computer Science and GIGA Research, University of Liège, Liège, Belgium; 3 L2S, Univ Paris Sud 11, Gif-sur-Yvette, France; Mount Sinai School of Medicine, United States of America

## Abstract

Fifty years ago, FitzHugh introduced a phase portrait that became famous for a twofold reason: it captured in a physiological way the qualitative behavior of Hodgkin-Huxley model and it revealed the power of simple dynamical models to unfold complex firing patterns. To date, in spite of the enormous progresses in qualitative and quantitative neural modeling, this phase portrait has remained a core picture of neuronal excitability. Yet, a major difference between the neurophysiology of 1961 and of 2011 is the recognition of the prominent role of calcium channels in firing mechanisms. We show that including this extra current in Hodgkin-Huxley dynamics leads to a revision of FitzHugh-Nagumo phase portrait that affects in a fundamental way the reduced modeling of neural excitability. The revisited model considerably enlarges the modeling power of the original one. In particular, it captures essential electrophysiological signatures that otherwise require non-physiological alteration or considerable complexification of the classical model. As a basic illustration, the new model is shown to highlight a core dynamical mechanism by which calcium channels control the two distinct firing modes of thalamocortical neurons.

## Introduction

Rooted in the seminal work of Hodgkin and Huxley [1], conductance-based models have become a central paradigm to describe the electrical behavior of neurons. These models combine a number of advantages, including physiological interpretability (parameters have a precise experimental meaning) and modularity (additional ionic currents and/or spatial effects are easily incorporated using the interconnection laws of electrical circuits [2], [3]). Not surprisingly, the gain in quantitative description is achieved at the expense of mathematical complexity. The dimension of detailed quantitative models makes them mathematically intractable for analysis and numerically intractable for the simulation of large neuronal populations. For this reason, reduced modeling of conductance-based models has proven an indispensable complement to quantitative modeling [4]–[6]. In particular, the FitzHugh-Nagumo model [7], a two-dimensional reduction of Hodgkin-Huxley model, has played an essential role over the years to explain the mechanisms of neuronal excitability (see e.g. [8], [9] for an excellent introduction and further references). More recently, Izhikevich has enriched the value of reduced-models by providing the Fitzugh-Nagumo model with a reset mechanism [10] that captures the fast (almost discontinuous) behavior of spiking neurons. Such models are used to reproduce the qualitative [11], [12] and quantitative [13], [14] behavior of a large family of neuron types. Notably, their computational economy makes them good candidates for large-scale simulations of neuronal populations [15].

The Hodgkin-Huxley model and all reduced models derived from it [7], [12] focus on sodium and potassium currents, as the main players in the generation of action potentials: sodium is a fast depolarizing current, while potassium is slower and hyperpolarizing. Initally motivated by reduced modeling of dopaminergic neurons in which calcium currents are essential to the firing mechanisms [16], the present paper mimicks the classical reduction of the Hodgkin-Huxley model augmented with an additional calcium current. The calcium current is a distinct player because it is depolarizing, as the sodium current, but acts on the slower timescale of the potassium current.

The inclusion of calcium currents in the HH model before its planar reduction leads to a novel phase portrait that seems to have been disregarded to date. Mimicking earlier classical work, we perform a normal form reduction of the global HH reduced planar model. The mathematical normal form reduction is fundamentally different in the classical and new phase portrait because it involves a different bifurcation. The classical fold bifurcation is replaced by a transcritical bifurcation.

The results of these mathematical analysis lead to a novel simple model that further enriches the modeling power of the popular hybrid model of Izhikevich. A single parameter in the new model controls the neuron calcium conductance. In low calcium conductance mode, the model captures the standard behavior of earlier models. But in high calcium conductance mode, the same model captures the electrophysiological signature of neurons with a high density of calcium channels, in agreement with many experimental observations. For this reason, the novel reduced model is particularly relevant to understand the firing mechanisms of neurons that switch from a low calcium-conductance mode to a high calcium-conductance mode. Because thalamocortical (TC) neurons provide a prominent example of such neurons, they are chosen as a proof of concept of the present paper, the benefits of which should extend to a much broader class of neurons.

## Results

### Planar Reduction of Hodgkin-Huxley Model Revisited in the Light of Calcium Channels

Calcium channels participate in the spiking pattern by providing, together with sodium channels, a source of depolarizing currents. In contrast to sodium channels whose gating kinetics are fast, calcium channels activate on a slower time-scale, similar to that of potassium channels [17]. As a consequence, their activation opposes the hyperpolarizing effect of potassium current activation, resulting in bidirectional modulation capabilities of the post-spike refractory period. We model this important physiological feature by considering the HH model [1] with an additional non-inactivating voltage-gated calcium current 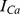 and a DC-current 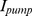 that accounts for hyperpolarizing calcium pump currents. The inactivation of calcium channels occurs in a slower time scale than the HH dynamics [18]. It can be modeled by a slower adaptation of the calcium conductance, which does not affect the single spike generation mechanism.

**Figure 1:**
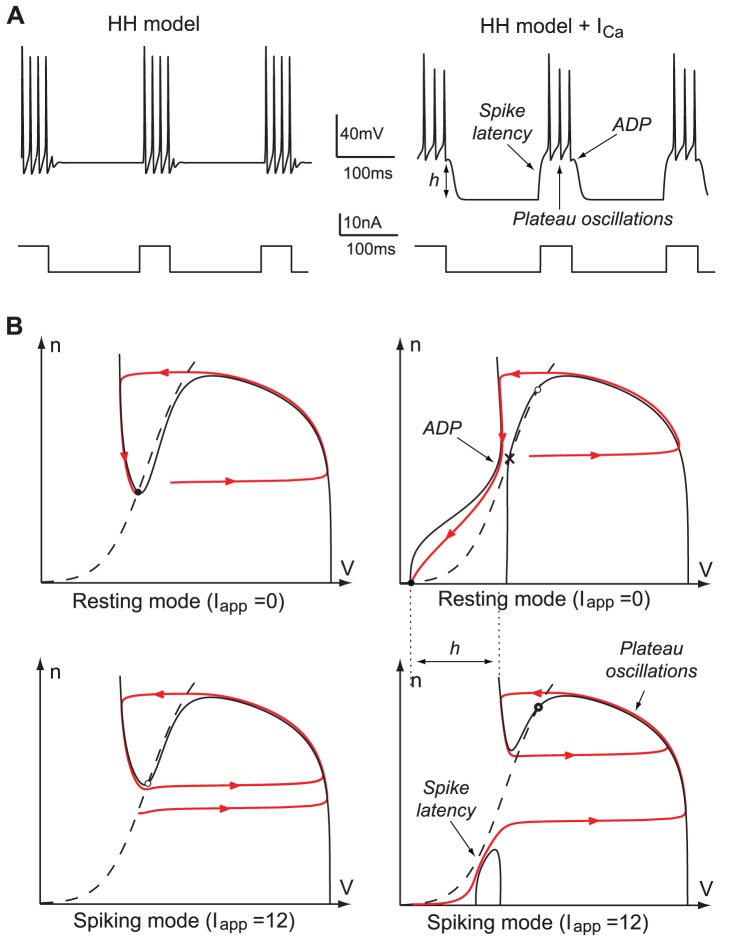
Step responses of the HH model without (left) and with a calcium current (right). (**A**) Time-evolution of the applied excitatory current (bottom) and of the corresponding membrane potential (top) in HH model (the reduced model leads to almost the same behavior (Fig. S1)). (**B**) Phase portraits of the reduced Hodgkin-Huxley model in resting (top) and spiking states (bottom). The 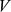
- and 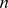-nullclines are drawn as a full and a dashed line, respectively. Trajectories are drawn as solid oriented red lines. Black circles denote stable fixed points, white circles unstable fixed points, and cross saddle points. The presence of calcium channels strongly affects the phase-portrait and the corresponding electrophysiological time-response of the neuron to excitatory inputs.

Figure 1 **A** illustrates the spiking behavior induced by the action of an external square current 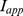 in the two different modes. As compared to the original HH model (Fig. 1 **A** left), the presence of the calcium current is characterized by a triple electrophysiological signature (see Fig. 1 **A** right):

- spike latency: the spike train (burst) is delayed with respect to the onset of the stimulation
- plateau oscillations: the spike train oscillations occur at a more depolarized potential than the hyperpolarized state
- after-depolarization potential (ADP): the burst terminates with a small depolarization

This electrophysiological signature is typical of neurons with sufficiently strong calcium currents. See for instance: spike latency [19], [20], plateau oscillations [21], ADPs [22], [23]. However, the mechanisms by which these behaviors occur have never been analyzed using reduced planar models to date.

Following the standard reduction of HH model [7], we concentrate on the voltage variable 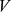 (that accounts for the membrane potential) and on a recovery variable 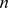 (that accounts for the overall gating of the ion channels) as key variables governing excitability (see methods). The phase-portrait of the reduced HH model is shown in Fig. 1 **B** (left). This phase portrait and the associated reduced dynamics are well studied in the literature (see [7] for the FitzHugh paper, and [9], [12] for a recent discussion and more references). We recall them for comparison purposes only. The resting state is a stable focus, which lies near the minimum of the familiar N-shaped 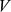-nullcline. When the stimulation is turned on (spiking mode), this fixed point loses stability in a subcritical Andronov-Hopf bifurcation (see below), and the trajectory rapidly converges to the periodic spiking limit cycle attractor. As the stimulation is turned off (resting mode), the resting state recovers its global attractivity via a saddle-node of limit cycles (the unstable one being born in the subcritical Hopf bifurcation), and the burst terminates with small subthreshold oscillations (cf. Fig. 1 **A** left).

In the presence of the calcium current, the phase-portrait changes drastically, as shown in Fig. 1 **B** (right). In the resting mode, the hyperpolarized state is a stable node lying on the far left of the phase-plane. The 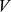-nullcline exhibits a “hourglass” shape. Its left branch is attractive and guides the relaxation toward the resting state after a single spike generation. The sign of 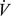 changes from positive to negative approximately at the funnel of the hourglass, corresponding to the ADP apex. The right branch is repulsive and its two intersections with the 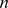-nullcline are a saddle and an unstable focus.

When the stimulation is turned on, the 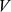-nullcline breaks down in an upper and a lower branch. The upper branch exhibits the familiar N-shape and contains an unstable focus surrounded by a stable limit cycle, very much as in the reduced Hodgkin-Huxley model. In contrast, the lower branch of the 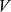-nullcline, which is not physiological without the calcium currents, comes into play. While converging toward the spiking limit cycle attractor from the initial resting state, the trajectory must travel between the two nullclines where the vector field has smaller amplitude. As a consequence, the first spike is fired with a latency with respect to the onset of the stimulation, as observed in Fig. 1 **A** (right) in the presence of the calcium current (see also Fig. S1). In addition, a comparison of the relative position of the resting state and the spiking limit cycle in Fig. 1 **B** (right) explains the presence of plateau oscillations. As the stimulation is turned off the spiking limit cycle disappears in a saddle-homoclinic bifurcation (see below), and the resting state recovers its attractivity.

**Figure 2:**
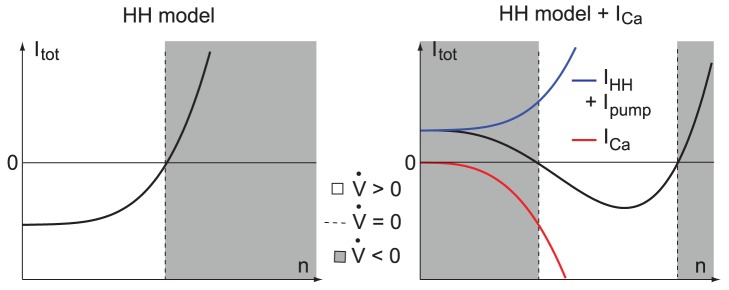
Total ionic currents for 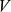 fixed as a function of 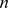 without (left) and with calcium channels (right). Blank portions corresponds to the values of 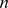 where 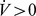, shaded portions corresponds to the values of 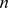 where 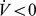, and the dashed lines correspond to the values of 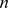 where 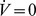. The hyperpolarizing calcium pump current 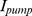 accounts for the vertical shift of the curve in the right figure. Note that the total ionic currents monotonically increase only in the absence of calcium channels (left).

The presence of the lower branch of the 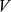-nullcline has a physiological interpretation. In the reduced HH model, the gating variable 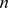 accounts for the activation of potassium channels and the inactivation of sodium channels. Their synergy results in a total ionic current that is monotonically increasing with 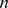 for a fixed value of 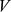 (Fig. 2, left). In this situation, at most one value of 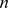 solves the equation 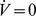 and there can be only one branch for the voltage nullcline. In contrast, when calcium channels are present, the reduced gating variable must capture two antagonistic effects. As a result, the total ionic current is decreasing for low 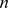 (the gating variable is excitatory), and increasing for large 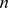 (the gating variable recovers its inhibitory nature) (Fig. 2, right). In this situation, two distinct values of 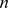 solve the equation 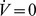, which explains physiologically the second branch of the 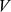-nullcline. To summarize, the lower branch of the voltage nullcline accounts for the existence of an excitatory effect of 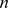, which is brought by calcium channel activation.

**Figure 3:**
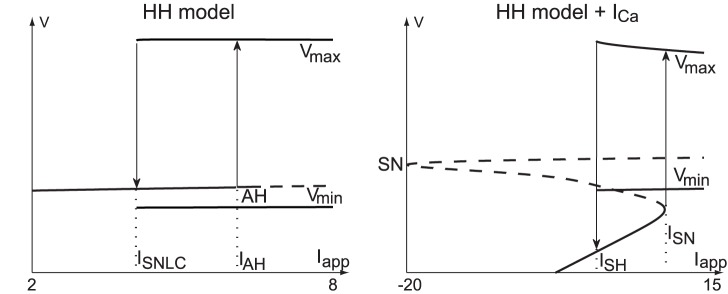
One parameter bifurcation diagram of the reduced Hodgkin-Huxley model without (left) and with calcium channels (right). Thin solid lines represents stable fixed points, while dashed lines unstable fixed points or saddle points. The thick lines labeled 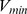 and 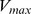 represent the minimum and the maximum voltage of stable limit cycles, respectively. 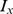, with 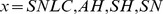, denotes the value of the input current for which the system undergoes the bifurcation 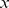.

A bifurcation diagram with 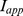 as the bifurcation parameter sheds more light on the transition mechanism between the resting and spiking modes (Fig. 3). We use XPPAUT [24] for this numerical analysis. We draw the bifurcation diagram without (left) and with calcium channels (right) only for small 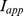, corresponding to the transition from resting to limit cycle oscillations (Fig. 3) (for larger 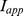, the stable limit cycle disappears in a supercritical Andronov-Hopf bifurcation in both cases, which leads to a stable depolarized, *i.e.* high-voltage, state).

Fig. 3 (left) illustrates the bifurcation diagram of the original reduced Hodgkin-Huxley model. For low values of 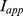, the unique fixed point is a stable focus that loses stability in a subcritical Andronov-Hopf bifurcation at 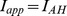. Beyond the bifurcation, the trajectory converges to the stable spiking limit cycle. When 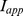 is lowered again below 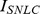, the spiking limit cycle disappears in a saddle-node of limit cycles, the unstable one (not drawn) emanating from the subcritical Andronov-Hopf bifurcation, and the trajectory relaxes back to rest.

Fig. 3 (right) illustrates the bifurcation diagram of the reduced Hodgkin-Huxley model in the presence of calcium channels. For 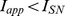, a stable node (lower branch), a saddle (central branch), and an unstable focus (upper branch) are present, as in Fig. 1 **B**(top right). The node and the saddle coalesce in a supercritical fold bifurcation at 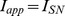, and disappear for 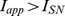, letting the trajectory converge toward the stable limit cycle. The spike latency observed in the 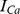-on configuration unmasks the ghost of this bifurcation. The stable limit cycle disappears in a saddle homoclinic bifurcation as 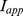 falls below 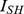, which lets the trajectory relax back to the hyperpolarized state. The homoclinic bifurcation exhibited by the Hodgkin-Huxley model with calcium channels is a key mathematical difference with respect to the standard HH model.

### The Central Ruler of Excitability is a Transcritical Bifurcation, not a Fold One

The power of mathematical analysis of the reduced planar model (1) is fully revealed by introducing two further simplifications.

- Time-scale separation: we exploit that the voltage dynamics are much faster than the recovery dynamics by assuming a small ratio 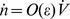 (the approximation holds away from the voltage nullcline) and by studying the singular limit 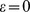.
- Transcritical singularity: by comparing the shape of the voltage nullcline in Fig. 1 **B**(right) for (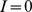) and (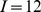), one deduces from a continuity argument that a critical value 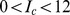 exists at which the two branches of the voltage nullcline intersect.

**Figure 4:**
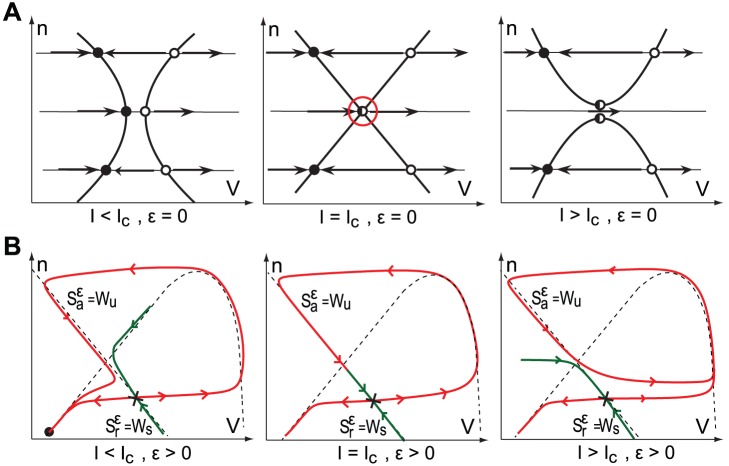
Transcritical bifurcation as the main ruler of neuronal excitability. (**A**) Cartoon of the V-nullcline transition through a singularly perturbed transcritical bifurcation. Black circles denote stable fixed points, white circles unstable fixed points. (**B**) Continuation of the stable 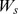 (in green) and the unstable 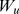 (in red) manifolds of the saddle away from the singular limit (*i.e.* 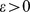). They dictate the transition from the resting state (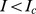) to the the spiking limit cycle (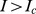) via a saddle-homoclinic bifurcation (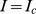).

The critical current 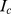 depends on 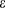. In the singular limit (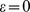) and for the corresponding critical current 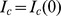, one obtains the highly degenerate phase portrait in Fig. 4 **A** (center). This particular phase portrait contains a transcritical bifurcation (red circle) which is the key ruler of excitability. This is because, as illustrated in Fig. 4 **B** for 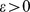, the convergence of solutions either to the resting point (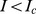) or to the spiking limit cycle (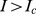) is fully determined by the stable 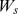 and unstable 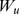 manifolds of the saddle point. In the singular limit shown in Fig. 4 **A**, these hyberbolic objects degenerate to a critical manifold that coincides with the voltage nullcline near the transcritical bifurcation. It is in that sense that the X-shape of the voltage nullcline completely organizes the excitability, i.e. the transition from resting state to limit cycle.

The persistence of the manifold 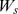 and 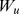 away from the singular limit can be rigorously established by geometric singular perturbation. The details of this analysis are available in the report [25]. The same analysis also establishes a normal form behavior in the neighborhood of the transcritical bifurcation: in a system of local coordinates centered at the bifurcation, the voltage dynamics take the simple form


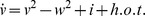


where 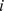 is a re-scaled input current and with 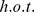 referring to higher order terms in 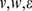.

It should be emphasized that it is the same perturbation analysis that leads to the classical view of the Hodgkin-Huxley reduced dynamics: the transition from Fig. 1 **B** left (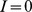) to Fig. 1 **B** left (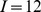) involves a fold bifurcation that governs the excitability with a fold normal form


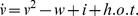


**Figure 5:**
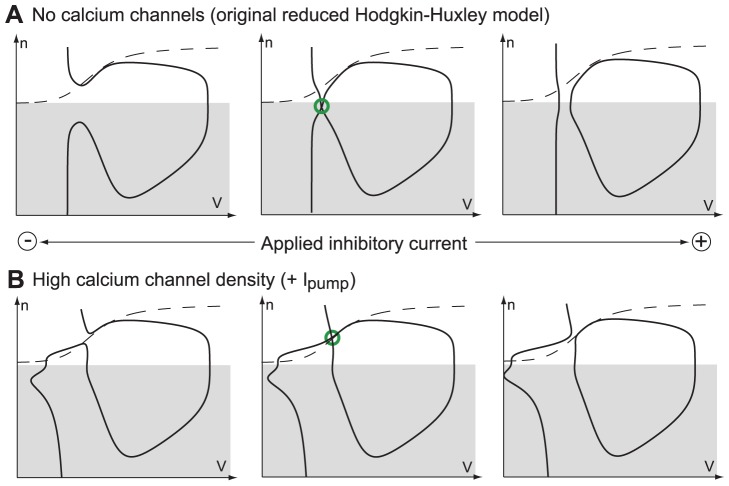
Unfolding of the transcritical bifurcation in the global reduced Hodgkin-Huxley phase portrait. (**A** and **B**) Phase portraits or the original reduced HH model without (**a**) and with a calcium current (**b**). The unshaded area represents the area of physiological relevance. A constant inhibitory current of increasing amplitude (from left to right) is applied to the model. The transcritical bifurcation (green circle) is non physiological in the classical reduced HH model (**A**) but plays an important physiological role in the presence of calcium channels (**B**).

It is of interest to realize that the addition of the calcium current in the HH model unmasks a global view of its phase portrait that has been disregarded to date for its lack of physiological relevance. Figure 5 **A** shows the phase portrait of the classical reduced HH model for three different values of the hyperpolarizing current, revealing the transcritical singularity for the middle current value. The unshaded part of the first plot (and only this part of the plot) is familiar to most neuroscientists since the work of FitzHugh. Likewise, the conceptual sketch of the transcritical bifurcation will be familiar to all readers of basic textbooks in bifurcation analysis. For instance, the sketch is found in [26] as a prototypical example of non-generic bifurcation. It is symptomatic that this particular example is described at length but not connected to any concrete model in a texbook that puts much emphasis on the relevance of bifurcation analysis in neurodynamics applications. As shown in Fig. 5 **B**, the missing connection is brought to life by calcium channels. Their particular kinetics renders the transcritical bifurcation of HH model physiological in the presence of a high-conductance calcium current.

### Transcritical Hybrid Modeling of Neurons

The singular limit of planar reduced models reveals that the excitability properties of spiking neurons are essentially determined by a local normal form of bifurcation of the resting equilibrium. This property is at the core of mathematical analysis of neuronal excitability (see [9], [12] and the rich literature therein).

In recent work, Izhikevich showed that, for computational purposes, the combination of the local normal form dynamics with a hybrid reset mechanism, mimicking the fast (almost discontinuous) spike down-stroke, is able to reproduce the behavior of a large family of neurons with a high degree of fidelity [10], [11]. Mimicking Izhikevich approach, we simplify the planar dynamics into the hybrid model:


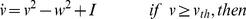



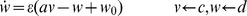


The proposed transcritical hybrid model is highly reminiscent of the hybrid model of Izhikevich, but it consideraly enlarges its modeling power by including two features of importance:

- the transcritical normal form 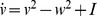 replaces the fold normal form 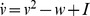, in accordance with the normal form analysis presented earlier.
- the new parameter 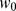 determines whether the intersection of the voltage and recovery nullclines will take place above (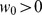) or below (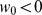) the transcritical singularity.

The parameter 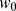 is a direct image of the calcium conductance: for small calcium conductances, the recovery variable nullcline only intersects the upper branch of the voltage nullcline (Fig. S2); likewise in the hybrid model when 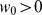. For high calcium conductance, the recovery variable nullcline intersects the lower branch of the voltage nullcline (Fig. S2); likewise in the hybrid model when 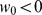.

**Figure 6:**
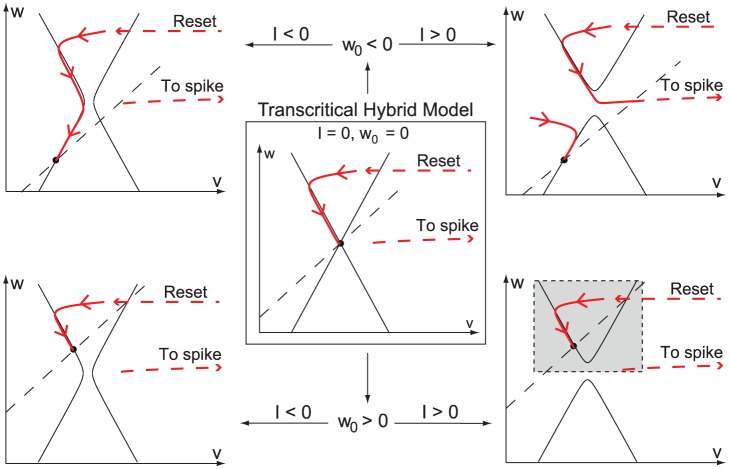
Schematic phase-portraits of the transcritical hybrid model for different values of 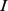 and 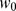. The 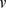
- and 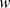-nullclines are drawn as full and dashed lines, respectively. The trajectories are drawn as red oriented lines. Many different phase-portraits derive from the transcritical hybrid model, including the one of the fold hybrid model, which only captures the shaded area.

Fig. 6 summarizes the four different phase portraits that derive from the transcritical hybrid model for different values of 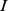 and 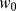. For 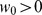, the model captures the classical view of the reduced HH model Fig. 6(bottom). For 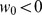, the model reveals the novel excitability properties associated to a high calcium conductance Fig. 6(top).The reader will notice the similarity between the phase portraits of Fig. 1 and of Fig. 6. The lower plots in Fig. 6 (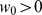) correspond to the left plots in Fig. 1 (HH model, no calcium current). Likewise, the upper plots in Fig. 6 (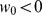) correspond to the right plots in Fig. 1 (HH model + calcium current).

### Reduced Modeling of a Thalamocortical Relay Neuron

**Figure 7:**
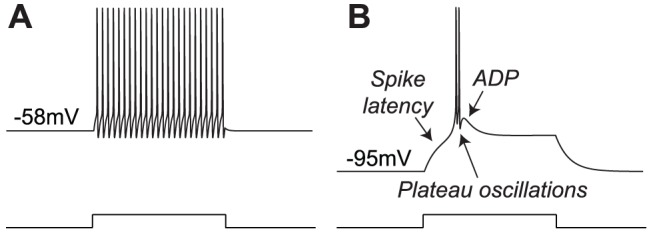
Step responses of a conductance-based TC neuron model (adapted from [**46**]) in low (left) and high calcium conductance modes (right). (**A** and **B**): Membrane potential variations of the simulated TC neuron over time in both conditions. The model reproduces the firing patterns exhibited by TC relay cells, namely tonic and burst firing.

Thalamocortical (TC) relay neurons are the input to sensory cortices. These neurons exhibit two distinct firing patterns: either a continuous regular spiking (Fig. 7 **A**) or a plateau burst spiking (Fig. 7 **B**) [27]–[30]. The switch between the two modes is regulated by prominent T-type calcium currents that are deinactivated by hyperpolarization, thereby modulating the resting membrane potential [28]–[32]. These firing patterns have been observed during both *in vitro* and *in vivo* recordings [33]–[37], and have been shown to play an important role in thalamocortical relay [38]–[41]. Among others, synchronous burst firing of TC relay cells is the key component of slow-wave sleep [40], [42], whereas the pathological generation of this firing pattern during wakefulness leads to absence epilepsy [43]–[45].

Because TC relay cells have an important role in physiology and pathology, they have been widely studied in the literature. In particular, their electrophysiological activity has been successfully reproduced in various conductance-based models [18], [29], [30], [46]–[48]. The two distinct spiking modes of TC neurons and their dependence on the activation of calcium channels make them a good candidate to test the relevance of our qualitative reduced model against quantitative conductance-based models. In order to verify this hypothesis, we perform a classical two-dimension reduction of a state-of-the-art conductance based model proposed in [46] (see Methods for the reduction details).

**Figure 8:**
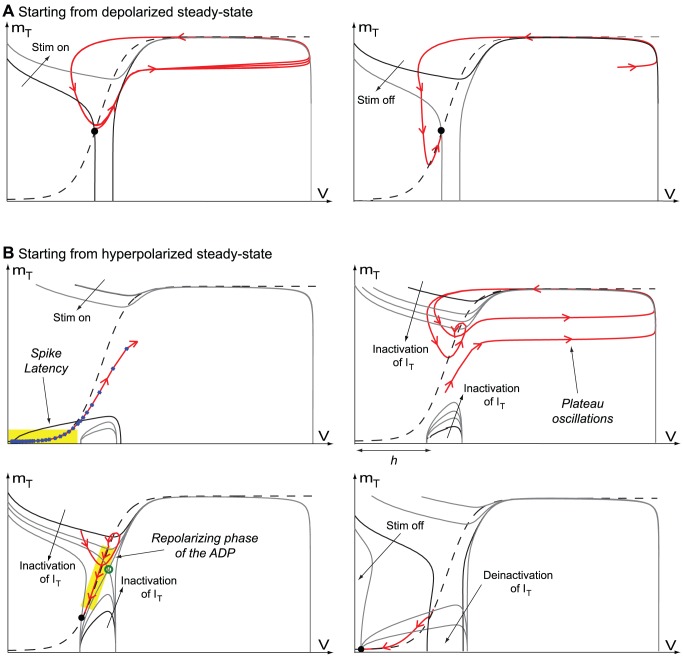
Phase-portraits of the reduced conductance based TC model in low (A) and high calcium conductance modes (B). The 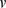
- and 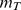-nullclines are drawn as full and dashed lines, respectively, and the hyperpolarized state as a filled circle 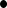. The trajectories are drawn as red oriented lines. In high calcium conductance mode, the phase portrait shows two 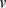-nullclines branches which derives from a transcritical singularity.

Fig. 8 shows the phase portraits of the reduced TC model in the two different modes. When the neuron is initially depolarized, T-type calcium channels are inactivated, and the phase portrait is similar to the traditional FitzHugh-Nagumo model: the voltage nullcline has a simple (upper) branch, which breaks into a left and a right branches when the stimulation is off (Fig. 8 **A**, black solid curves). The accompanying transcritical singularity appears at non-physiological values of the gating variable 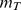. When the stimulation is turned on, the 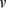-nullcline upper branch rises (Fig. 8 **A**, light gray solid curve), the fixed point looses its stability to a limit cycle that relaxes to the resting state when the stimulation is switched off. Likewise, no plateau is observed and the repolarization phase is strictly monotonic (no ADP can be exhibited).

The situation is very different when the neuron is initially hyperpolarized, because T-type calcium channels are then deinactivated, critically affecting the phase portrait: a lower 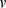-nullcline branch is now present for physiological values of 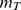, and the hyperpolarized state belongs to this lower branch (Fig. 8 **B**, upper-left, black gray solid curves). When a depolarizing current step is applied, this branch falls below the 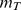-nullcline (Fig. 8 **B**, upper-left, light gray solid curves). In order to generate the first spike, the state travels the narrow region between the two nullclines, resulting in a pronounced latency. This latency relies on the small level of 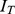 activation in this hyperpolarized state (the 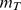-nullcline is almost horizontal), and is amplified by the dynamic inactivation of T-type calcium channels, which further narrows this funnel. This observation in agreement with previous experimental and modeling data [48]. Furthermore, the relative position of the hyperpolarized state (lower branch) with respect to spiking cycle (upper branch) clearly explains the generation mechanism of plateau oscillations.

These high frequency plateau oscillations continue until the T-type calcium channel inactivation dominates (Fig. 8 **B**, upper-right). At the end of the burst, the system converges toward the hyperpolarized state, being attracted first by the upper part of the 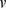-nullcline (depolarizing phase of the ADP), then by its lower part (repolarizing phase of the ADP) (Fig. 8 **B**, lower-left). Note the presence of a transcritical bifurcation in the phase portrait for a particular value of T-type calcium channel inactivation (Fig. 8 **B**, lower-left, green circle). Finally, when the stimulation is relaxed, the neuron recovers its initial hyperpolarized state and the inactivation of T-type calcium channels is released (Fig. 8 **B**, lower-right).

This phase portrait analysis confirms that the transcritical singularity in indeed a key ruler of excitability in this reduced conductance based TC neuron model. As a consequence, our proposed transcritical hybrid model seems appropriate to capture the essence of its firing mechanisms.

### Transcritical Hybrid Modeling of a Thalamocortical Relay Neuron

The phase portrait analysis in the previous section suggests the relevance of a reduced transcritical hybrid model to model TC relay neurons. We emphasize that our objective is not a fine tuned quantitative modeling of the TC neuron firing pattern. Rather, we attempt to provide a qualitative picture of how the proposed simple hybrid dynamics permits to reproduce and explain the behavior of TC neurons and, in particular, the role of calcium currents.

**Figure 9:**
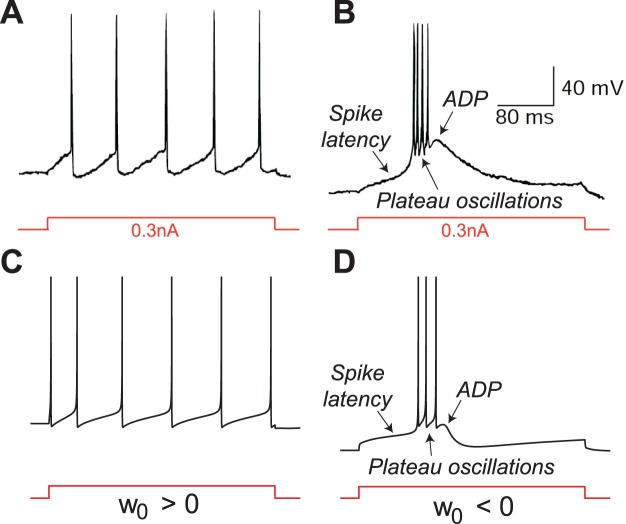
Comparison of the experimental step response of a TC neuron in vitro [**41**] (top) and the step response of the proposed transcritical hybrid model (bottom) in low (left) and high calcium conductance modes (right). (**A** and **B**): Membrane potential variations of the recorded TC neuron over time in both conditions. (**C** and **D**) Membrane potential variations of the modeled TC neuron over time in both conditions. A variation of 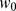, which is an image of the calcium conductance, is sufficient to generate the switch of firing pattern physiologically observed in TC cells.

Fig. 9 compares the experimental step response of a TC neuron *in vitro* and the simulated step response of the transcritical hybrid model (1), both in the low and high calcium conductance modes. As discussed above, the small calcium conductance mode is obtained by choosing a positive 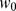 (T-type calcium channels are inactivated), whereas the large calcium conductance mode is obtained by choosing a negative 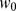 (T-type calcium channels are deinactivated), all the other parameters being identical in the two modes. An additional variable 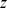 accounts for the slow adaptation mechanisms, such as e.g. T-type calcium channels inactivation and variations of intracellular calcium concentration. The hybrid model reproduces the experimental observation: in the low-calcium mode, it responds with a slow regular train of action potentials; in the high-calcium mode, it responds with a long spike latency, plateau oscillations, and an ADP.

**Figure 10:**
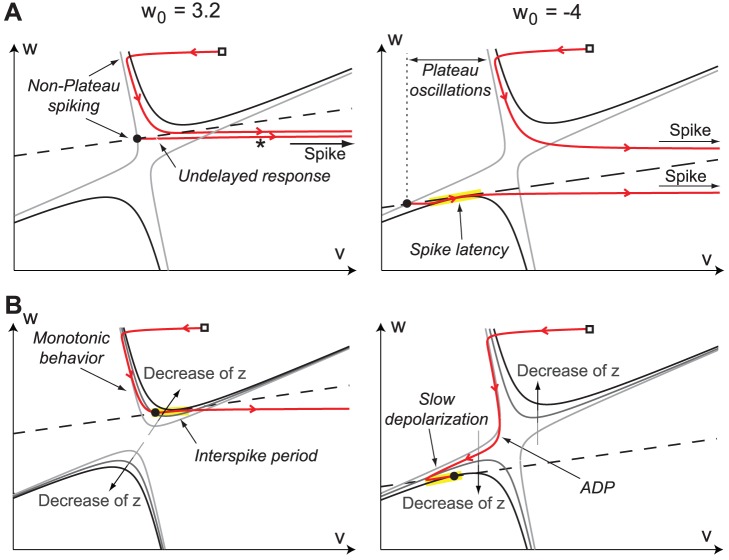
Phase-portrait of the transcritical hybrid model of a TC cell in the low (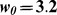, left) and high calcium conductance modes (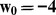, right). Trajectories are depicted as solid oriented red lines. The reset point is depicted as a square 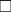, while the (instantaneous) hyperpolarized state as a filled circle 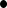. The 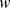-nullcline is depicted as a dashed line. In (**A**), the gray (black) thin solid line is the 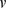-nullcline when the current step in off (on). In (**B**), the 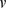-nullcline is depicted as gray thin lines of different darkness. As sketched in the figure, light gray correspond to large values of 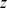, whereas dark gray to small.

Fig. 10 shows the phase-portraits of the transcritical hybrid model in the two modes. Note the great similarity with these phase portraits and the ones of the reduced conductance-based TC neuron model. When 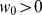 (Fig. 10, left), the hyperpolarized state belongs to the upper branch of the 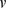-nullcline. Application of a depolarizing current step lifts the voltage nullcline above the resting state, thus generating a transient non-delayed action potential (marked with a 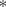 in Fig. 10 **A**, left). When the hyperpolarized state belongs to the upper branch of the 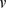-nullcline, no plateau oscillations are possible (Fig. 10 **A**, left). Furthermore, the relaxation toward the hyperpolarized state is necessarily monotone (*i.e.* no ADP), as stressed in Fig. 10 **B**, left.

At the generation of the first spike, 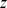 increases, which reduces the excitability of the cell (calcium accumulates in the intracellular space for instance, which activates hyperpolarizing calcium pumps), and the 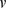-nullcline upper branch falls. As the neuron remains polarized, 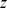 decreases (the intracellular calcium is expelled), calcium pump currents slowly deactivate and the cell slowly depolarizes (interspike period), until the spiking threshold is reached and a new action potential is fired (Fig. 10 **B**, left).

When 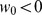 (Fig. 10, right), the hyperpolarized state belongs to the lower branch of the 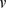-nullcline, and is more hyperpolarized than for 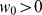, as in experiments. When a depolarizing current step is applied, this branch falls below the 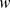-nullcline (Fig. 10 **A**, right). In order to generate the first spike, the state travels in the narrow region between the two nullclines, resulting in a pronounced latency. Furthermore, the relative position of the hyperpolarized state (lower branch) with respect to (hybrid) spiking cycle (upper branch) clearly explains the generation mechanism of plateau oscillations, as in the reduced TC model. These high frequency plateau oscillations (burst) continue until 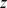 is sufficiently large (T-type calcium channels are inactivated and calcium accumulates in the cytoplasm). Plateau oscillations then terminate in a (hybrid) saddle-homoclinic bifurcation (Fig. 10 **A**, right).

At the end of the burst, the system converges toward the hyperpolarized state following the left branch of the 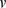-nullcline, thus generating a marked ADP at the passage near the nullcline funnel. The subsequent slow phase is mainly ruled by the variations of the intracellular calcium. With the adopted simple dynamics it consists in a slow depolarization that follows the decrease of the intracellular calcium (Fig. 10 **B**, right). A finer and more physiological modeling of the intracellular calcium dynamics could reproduce *in vitro* recordings with a higher degree of fidelity. Note that the ADP trajectories are slightly different in the reduced conductance based and the transcritical hybrid models. This minor difference is due to a lower time-scale separation in the reduced TC model for low values of 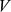, but the generating mechanisms are similar. We discuss the impact of this difference in the next section.

In order to further verify the physiological consistence of the transcritical model, we compare its behavior with the simulated step response of another quantitative one compartment model [29], [30], a quantitative 200-compartments model [46] (simulations were run in the *Neuron* environment, based on the configuration files freely available at http://cns.iaf.cnrs-gif.fr/alain_demos.html) and a fold hybrid model [12] of a TC relay cell in the large conductance mode (Fig. S3). For the quantitative models, we plot the trajectory projection on the 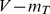 plane, where 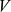 and 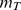 denotes the somatic membrane potential and the activation gating variable of the somatic T-type calcium current, respectively.

There is a striking similarity between the projection of the trajectories between both quantitative models and the phase portrait of the second-order transcritical model. In both cases, the ADP is generated during a decrease of the activation variable, and plateau oscillations are exhibited far from the resting state. Moreover, the spike latency is a robust property of the transcritical model because the trajectory must visit the neighborhood of both the nullclines 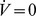 and 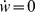 before converging to the spiking limit cycle. It should be stressed that there are no comparable ways to reproduce this behavior in a fold hybrid model. Indeed, as highlighted above, reproducing this behavior with the standard reduced HH model necessitates a non physiological alteration of the reset rule (Fig. S3 [12], [49]). This underlines the relevance of the revisited model to capture the richness of neuronal excitability.

### Robust ADP Generation

**Figure 11:**
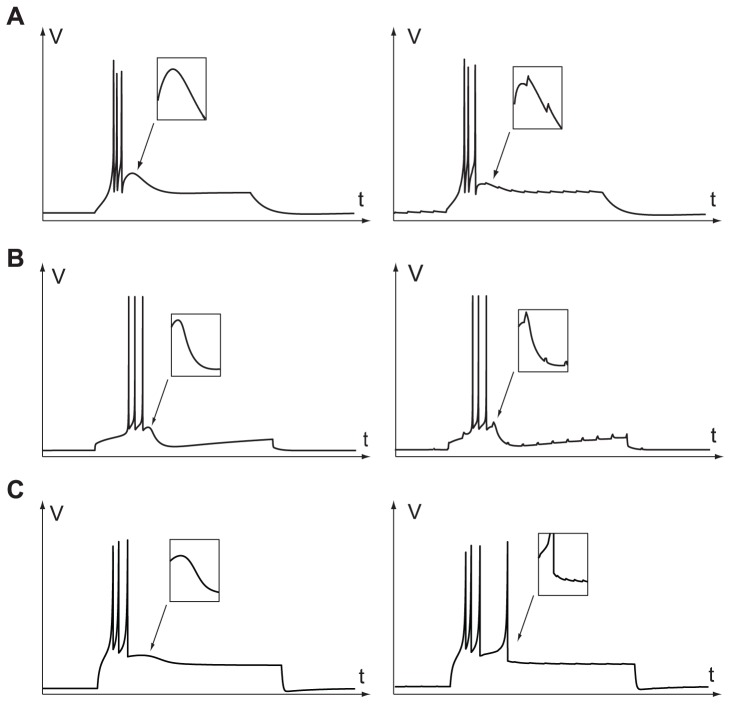
Nominal step response (left) and step response in the presence of small current pulses in the 200 compartments TC neuron model (A), the transcritical hybrid model (B), and the fold hybrid model of TC neuron ([**11**]) (C).

For a neuron model to be biologically relevant, it should be robust to exogenous disturbances (small synaptic inputs, thermal noise, etc.). The firing pattern, in particular, should remain unchanged. Fig. 11 compares the perturbation robustness of three TC neuron models to small current impulses. It suggests that the fold hybrid model is less robust than the transcritical model, because a tiny pulse is sufficient to generate an extra action potential at the ADP apex.

**Figure 12:**
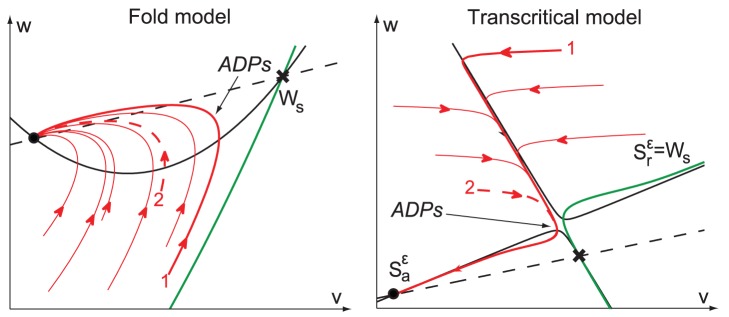
Comparison of the ADP generation mechanisms in the fold (left) and in the transcritical hybrid models (right). The stable manifold of the saddle (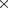) is depicted in green. In the fold hybrid model, ADPs are generated by sliding near the stable manifold of the saddle and crossing the 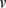-nullcline from below. In the transcritical hybrid model, ADPs are robustly generated along the attractor 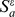.

The difference in robustness is explained by the different ADP generation mechanisms, as illustrated in Figure 12. In the fold model, ADPs are generated when trajectories cross the 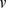-nullcline from below (Fig. 12, see also Fig. S3). The absence of any robust attractor in the ADP generation region makes the ADP height and shape heavily dependent on the exact reset point. Moreover, when small current pulses are applied, the ADP generation is disrupted, and the model fires an extra (non-physiological) spike.

Conversely, ADP generation in the transcritical hybrid model is robustly governed by the attractor 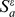 that steers the trajectories through the ADP apex and toward the resting point. That is the reason why the ADP height and shape barely depend on chosen reset point. At the same time, the persistence to small perturbations of this invariant manifold [50] ensures, as required in biologically meaningful conditions, the robustness of the ADP generation mechanism to small inputs.

The ADP apex is the most excitable part of the trajectory. A physiologically relevant external stimulation can easily generate a spike during this small period, whereas the neuron is barely excitable in the preceding and following periods. This suggests a major role for this ADP in the modulation of external inputs by neuron endogenous rhythm. This excitability can be finely tuned through variations of channel kinetics. For instance, the presence of a “buckle” in the ADP trajectory of the reduced conductance based model in Fig. 8 (compare to Fig. S3) is an artifact that illustrates the sensitivity trajectories around the ADP apex, a mathematical illustration of the neuron excitability at this particular instant.

## Discussion

### Calcium Channels Physiologically Unmask the Physiological Relevance of a Global View of the Reduced Hodgkin-Huxley Phase Portrait

The inclusion of calcium channels in Hodgkin-Huxley model has a dramatic impact on its mathematical reduction: the firing mechanisms are governed by the local normal form of a transcritical rather than fold bifurcation. It results in the presence of a new voltage nullcline branch in the phase portrait, which lies below the classical inverted N-shaped one. When the calcium channel density is high, neuronal excitability is governed by this lower branch, which accounts for the physiological signature of these currents: spike latency, plateau oscillations and afterdepolarization potential.

Interestingly, it is not the phase portrait of the reduced HH model that is affected by calcium, but only the subregion of the plane where it is physiologically relevant. Indeed, the transcritical singularity is the core mechanism of the classical Hodgkin-Huxley model as well, and the well known fold bifurcation derives from it. As a consequence, the classical FitzHugh Nagumo phase portrait is a particular (because localized) view of the more complete picture studied in the present paper. This complete picture and its dynamical consequences are brought to live by any slowly activating depolarizing current, calcium currents being the most representative. Note that it may be generated by a slowly activating persistent sodium current as well.

### The Proposed Planar Model Differs from Earlier Planar Models that Include Calcium Channels

The proposed planar model is distinctively different from earlier reduced models that include calcium channels in that its slow variable (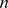 or 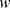) aggregates in the same time scale the antagonistic activation of potassium and calcium channels, thereby capturing the non monotonicity of the total ionic current (Fig. 2, right). This property is lost when the activation of calcium channels is treated as a fast variable, that is, set at steady-state in the reduction such as, for instance, in the popular Morris-Lecar model [51]. A classical reference such as [17] nevertheless suggests that the time constant of calcium and potassium activations are comparable, motivating the reduction adopted in the present paper.

### The Richness of Neuronal Excitability is Captured in a Two Dimensional Transcritical Hybrid Model

Although this enlarged phase portrait is the source of rich and diverse forms of excitability, its essence is captured in a simple and physiologically grounded hybrid model. The illustration of its modeling power on the thalamocortical neuron excitability shows the impact of revisiting the classical view. Indeed, whereas tonic spiking of these cells is well captured by classical models based on the fold normal form, the generation of burst firing needs non physiological alterations of the phase portrait and the reset rule of these models. On the other hand, both firing patterns can be generated in the transcritical hybrid model through a change in one parameter, which reflects the proportion of calcium channels which are not inactivated.

This illustration is just the top of the iceberg because the same principle will apply to many important families of neurons that are thoroughly studied and that have so far largely resisted reduced modeling. The proposed model will impact the understanding of excitability of e.g. dopaminergic, serotonergic, and subthalamic nucleus neurons, whose various firing patterns have a direct and critical impact in physiology and diseases, such as Parkinson’s disease and depression.

### A Transcritical Hybrid Model as the Basic Unit of Large Population Studies

Detailed modeling of large scale neuronal networks is an invaluable tool in the analysis of the mechanisms underlying the collective behavior of the brain. The recent paper [15] illustrates that reduced models such as those discussed here are simple enough to be used in large-scale population computations including tens of thousands of neurons. Because the proposed transcritical hybrid model captures a broader class of neuronal dynamics and is particularly adapted to neurons expressing a high density of calcium channels, it will be an ideal candidate for physiologically realistic studies of high-dimensional neuronal networks such as the thalamocortical circuitry or the basal ganglia motor loop.

## Materials and Methods

### Equation and Parameters of the Complete Model

The augmented HH model reads.


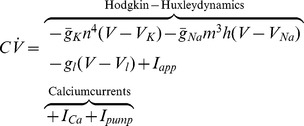



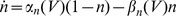



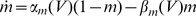



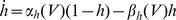


For the HH dynamics, we use the parameters of the original paper [1]. As all other HH currents, the additional calcium current obeys Ohm’s law.


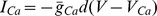


where 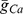 is the maximum calcium conductance, 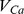 is the calcium Nernst potential, and 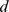 is the calcium activation gating variable. In our study, we consider the kinetics of L-type calcium channels (adapted from [3]):


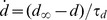



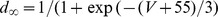



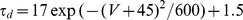


L-type calcium channels are calcium permeable channels that activate at high-threshold and are found in many cell types, such as dopaminergic or serotoninergic neurons, muscle and cardiac cells. The functions 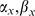, 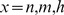, can be found in the paper [1]. The value for the potassium Nernst potential 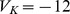 is the same as in [1], while the sodium Nersnt and the leak Nernst potential are rounded to 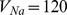 and 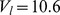, respectively. The values of the sodium 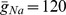, potassium 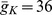, and leak 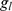 (maximum) conductances are the same as in [1]. The calcium Nernst potential is given by 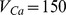. The numerical simulations of Fig. 1(right) are obtained by taking 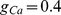 and 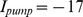.

### Planar Reduction and Phase Portrait Analysis

We follow the standard reduction of the original HH model to a two dimensional system by: i) assuming an instantaneous sodium activation, 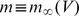, where 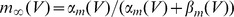; ii) exploiting the approximate linear relation, originally proposed in [7], 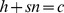, with 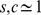. iii) exploiting the correlation between the potassium and calcium gating kinetics to approximate the calcium activation gating variable 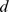 as a static function of the potassium activation gating variable 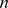 (the simple relationship 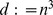 provides a satisfactory fit, see Fig. S4). Applying this reduction to (1) with parameters as above, we obtain the planar system (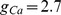).


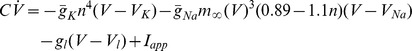



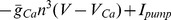



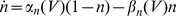



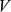
-“nullcline” refers to the set 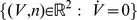 and similarly for other variables.

### Reduced Model of TC Neurons

The complete TC neuron model reads (adapted from [46])


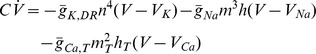



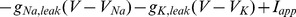



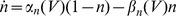



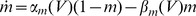



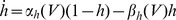



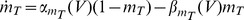



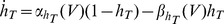


The dynamics of the ionic currents are similar to [46]. The conductance values are taken as follows (in 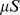): 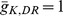, 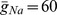, 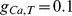, 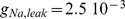 and 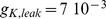. The membrane capacitance 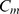 is set to 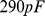. Holding currents are 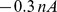 for bursting mode, and 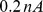 for tonic mode. The amplitude of the current steps are 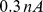 and 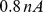 in bursting and tonic modes, respectively.

The reduction of the model is performed by assuming an instantaneous sodium activation 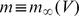 and merging 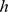, 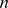 and 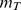 so that they have similar time courses in the range of interest. The reduced model reads.


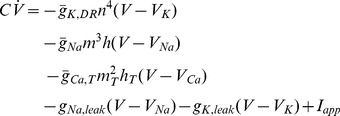



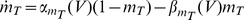



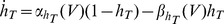


For the phase plane analysis, inactivation of T-type calcium channels 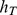 is considered as a parameter, its timescale being much slower than the other variables. To ensure a similar resting potential in both models, the holding current of the reduced model is adjusted to 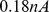 in tonic mode. The reduced model finely reproduces the behavior of the complete model in our range of interest (Fig. S5).

### Hybrid Modeling of TC Neurons

For modeling convenience, we add a fitting parameter 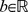 in the sub-threshold (continuous) voltage dynamics:


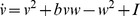


The extra parameter does not affect the nature of the bifurcation, but tunes the slope of the 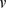-nullcline branches. In order to account for the effect of intracellular calcium variations and for the associated activation of calcium pump currents, we also add a slow adaptation variable 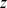 as follows:


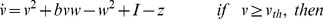



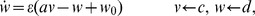



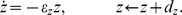


with 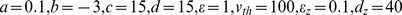. The low-calcium mode correspond to 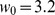, the high-calcium mode to 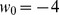. The injected current 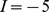 when the stimulation is off and 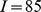 when the stimulation is on. We stress two properties that are not captured by the reduced model (1): Firstly, the slow adaptation variable (*i.e.* 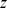) dynamics are generally coupled with the membrane voltage also in the subthreshold phase. Secondly, depending on the type of the modeled calcium current, the calcium conductance, here reflected by 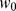 (Section), might slowly change in response to voltage and intracellular calcium variations. Both properties are neglected in (1).

### Numerical Simulations

Numerical simulations were run with MATLAB. (http://www.mathworks.com), apart from the 200-compartment model, which was simulated with the NEURON software environment. (http://www.neuron.yale.edu). The numerical bifurcation analysis has been obtained with XPP environment. (http://www.math.pitt.edu/ 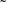
bard/xpp/xpp.html).

## Supporting Information

Figure S1 Stimulation-induced bursting in the reduced Hodgkin-Huxley model without (left) and with calcium current (right).
(EPS)

Figure S2
**Nullcline intersections in the reduced model (5) with calcium current for different values of the calcium conductance.** The 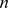-nullcline is depicted as a dashed line, the 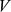-nullcline as a solid line. The associated calcium conductance is expressed via a gray scale, as indicated in the figure legend. The calcium pump currents is given by 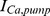 = 0.0, −0.74, −2.78, −15.6 for, respectively, 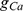 = 0.0, 1.0, 1.5, 3.0.(EPS)

Figure S3
**Comparison of the step response and phase-portrait in the transcritical hybrid model, a quantitative one compartment model of TC neuron** [**29]**, [**30**] **, a 200-compartment model of TC neurons** [**46**] **and the fold hybrid model** [**12**] **in large calcium conductance mode.** In the phase-portrait of the hybrid models, 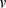
- and 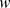-nullclines are drawn as full and dashed lines, respectively, and trajectories are drawn as red oriented lines. The black full line represents the 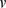-nullcline at the onset of the stimulation. The gray full line represents the 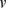-nullcline at the end of the burst. The phase-portrait of the conductance based models depicts the trajectory projection on the 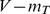 plane, where 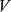 and 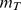 denotes the somatic membrane potential and the activation gating variable of the somatic T-type calcium current, respectively.(EPS)

Figure S4
**Time-courses of the calcium activation gating variable 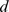 versus the potassium activation gating variable 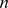 at the initiation of a burst.** The black line shows the 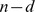 relationship in the complete model, the red line plots the relationship *d* = 1.5 *n* ^3^. The calcium activation gating variable is well approximated as a static function of the potassium activation gating variable in this region of interest.(EPS)

Figure S5 **Comparison of the complete and reduced conductance based models of TC relay cells, in tonic and bursting modes.** Black lines show the complete model step responses in both modes. Red lines show the reduced model step responses in both modes. The complete and reduced models has similar kinetics in our range of interest. Note that the reduced model shows an artifact at the apex of the ADP in bursting mode.
(EPS)

## References

[1] Hodgkin A, Huxley A (1952) A quantitative description of membrane current and its application to conduction and excitation in nerve. J. Physiol. 117: 500–544. 10.1113/jphysiol.1952.sp004764 PMC1392413 12991237

[2] Halnes G, Augustinaite S, Heggelund P, Einevoll GT, Migliore M (2011) A multi-compartment model for interneurons in the dorsal lateral geniculate nucleus. PLoS Comput. Biol. 7: e1002160. 10.1371/journal.pcbi.1002160 PMC3182861 21980270

[3] Canavier C, Landry R (2006) An increase in AMPA and a decrease in SK conductance increase bursting by different mechanisms in a model of a dopamine neuron in vivo. J Neurophysiol. 96: 2549–2563. 10.1152/jn.00704.2006 PMC2531289 16885519

[4] Rinzel J (1985) Excitation dynamics: insights from simplified membrane models. Fed Proc. 44: 2944–2946. 2415401

[5] Krinskii VI, Kokoz IuM (1973) [Analysis of the equations of excitable membranes. I. Reduction of the Hodgkins-Huxley equations to a 2d order system]. Biofizika. 18: 506–511. 4717781

[6] Kokoz IuM, Krinskii VI (1973) [Analysis of the equations of excitable membranes. II. Method of analysis of the electrophysiological characteristics of a Hodgkin-Huxley membrane from graphs of a 2d-order null-isocline system]. Biofizika. 18: 878–885. 4751866

[7] FitzHugh R (1961) Impulses and physiological states in theoretical models of nerve membrane. Biophys J. 1: 445–466. 10.1016/s0006-3495(61)86902-6 PMC1366333 19431309

[8] Rinzel J, Ermentrout GB (1989) Analysis of neural excitability and oscillations, 135–169. MIT Press, Cambridge, MA, USA.

[9] Ermentrout GB, Terman DH (2010) Mathematical Foundations of Neuroscience. Interdisciplinary Applied Mathematics. Springer.

[10] Izhikevich EM (2003) A simple model of spiking neurons. IEEE Trans Neural Netw. 14: 1569–1572. 10.1109/TNN.2003.820440 18244602

[11] Izhikevich EM (2010) Hybrid spiking models. Phil. Trans. R. Soc. 368: 5061–5070. 10.1098/rsta.2010.0130 20921012

[12] Izhikevich EM (2007) Dynamical Systems in Neuroscience: The Geometry of Excitability and Bursting. MIT Press.

[13] Pospischil M, Piwkowska Z, Bal T, Destexhe A (2011) Comparison of different neuron models to conductance-based post-stimulus time histograms obtained in cortical pyramidal cells using dynamic-clamp in vitro. Biol Cybern. 105: 167–180. 10.1007/s00422-011-0458-2 21971968

[14] Richert M, Nageswaran J, Dutt N, Krichmar J (2011) An efficient simulation environment for modeling large-cale cortical processing. Front Neuroinform. 5: 19. 10.3389/fninf.2011.00019 PMC3172707 22007166

[15] Izhikevich E, Edelman G (2008) Large-scale model of mammalian thalamocortical systems. Proc Natl Acad Sci U S A. 105: 3593–3598. 10.1073/pnas.0712231105 PMC2265160 18292226

[16] Drion G, Massotte L, Sepulchre R, Seutin V (2011) How modeling can reconcile apparently discrepant experimental results: The case of pacemaking in dopaminergic neurons. PLoS Comput. Biol. 7, e1002050. 10.1371/journal.pcbi.1002050 PMC3102759 21637742

[17] Hille B (1991) Ionic Channels of Excitable Membranes. Sinauer, Sunderland, Massachusetts, 2nd. edition.

[18] Wang XJ, Rinzel J, Rogawski MA (1991) A model of the T-type calcium current and the low-threshold spike in thalamic neurons. J Neurophysiol. 66: 839–850. 10.1152/jn.1991.66.3.839 1661326

[19] Rekling JC, Feldman JL (1997) Calcium-dependent plateau potentials in rostral ambiguus neurons in the newborn mouse brain stem in vitro. J Neurophysiol. 78: 2483–2492. 10.1152/jn.1997.78.5.2483 9356399

[20] Molineux ML, Fernandez FR, Mehaey WH, Turner RW (2005) A-Type and T-Type currents interact to produce a novel spike latency-voltage relationship in cerebellar stellate cells. J Neurosci. 25: 10863–10873. 10.1523/JNEUROSCI.3436-05.2005 PMC6725871 16306399

[21] Beurrier C, Congar P, Bioulac B, Hammond C (1999) Subthalamic nucleus neurons switch from single-spike activity to burst firing mode. J Neurosci. 19: 599–609. 10.1523/JNEUROSCI.19-02-00599.1999 PMC6782207 9880580

[22] Azouz R, Jensen MS, Yaari Y (1996) Ionic basis of spike after-depolarization and burst generation in adult rat hippocampal CA1 pyramidal cells. J Physiol. 49: 211–223. 10.1113/jphysiol.1996.sp021302 PMC1158874 8730596

[23] Chen S, Yaari Y (2008) Spike Ca2+ influx upmodulates the spike afterdepolarization and bursting via intracellular inhibition of KV7/M channels. J Physiol. 586: 1351–1363. 10.1113/jphysiol.2007.148171 PMC2375679 18187471

[24] Ermentrout GB (2002) Simulating, analyzing, and animating dynamical systems: a guide to XPPAUT for researchers and students. SIAM Press, Philadelphia, PA, USA.

[25] Franci A, Drion G, Seutin V, Sepulchre R (2011) A Novel Phase Portrait to Understand Neuronal Excitability. arXiv. 1112.2588v1. 10.1371/journal.pone.0041806 PMC3414513 22905107

[26] Seydel R (2010) Practical bifurcation and stability analysis, volume 5 of Interdisciplinary Applied Mathematics. Springer-Verlag, New York, third edition.

[27] Jahnsen H, Llinas R (1984) Electrophysiological properties of guinea-pig thalamic neurones: an in vitro study. J Physiol. 349: 205–226. 10.1113/jphysiol.1984.sp015153 PMC1199334 6737292

[28] Jahnsen H, Llinas R (1984) Ionic basis for the electro-responsiveness and oscillatory properties of guinea-pig thalamic neurones in vitro. J Physiol. 349: 227–247. 10.1113/jphysiol.1984.sp015154 PMC1199335 6737293

[29] McCormick DA, Huguenard JR (1992) A model of the electrophysiological properties of thalamocortical relay neurons. J Neurophysiol. 68: 1384–1400. 10.1152/jn.1992.68.4.1384 1331356

[30] Huguenard JR, McCormick DA (1992) Simulation of the currents involved in rhythmic oscillations in thalamic relay neurons. J Neurophysiol. 68: 1373–1383. 10.1152/jn.1992.68.4.1373 1279135

[31] Crunelli V, Lightowler S, Pollard CE (1989) A T-type Ca2+ current underlies low-threshold Ca2+ potentials in cells of the cat and rat lateral geniculate nucleus. J Physiol. 413: 543–561. 10.1113/jphysiol.1989.sp017668 PMC1189115 2557441

[32] Zhou Q, Godwin DW, O’Malley DM, Adams PR (1997) Visualization of calcium influx through channels that shape the burst and tonic firing modes of thalamic relay cells. J Neurophysiol. 77: 2816–2825. 10.1152/jn.1997.77.5.2816 9163395

[33] Guido W, Lu SM, Sherman SM (1992) Relative contributions of burst and tonic responses to the receptive field properties of lateral geniculate neurons in the cat. J Neurophysiol. 68: 2199–2211. 10.1152/jn.1992.68.6.2199 1491266

[34] Guido W, Lu SM, Vaughan JW, Godwin DW, Sherman SM (1995) Receiver operating characteristic (ROC) analysis of neurons in the cat’s lateral geniculate nucleus during tonic and burst response mode. Vis Neurosci. 12: 723–741. 10.1017/s0952523800008993 8527372

[35] Guido W, Weyand T (1995) Burst responses in thalamic relay cells of the awake behaving cat. J Neurophysiol. 74: 1782–1786. 10.1152/jn.1995.74.4.1782 8989413

[36] Sherman SM, Guillery RW (1996) The functional organization of thalamocortical relays. J. Neurophysiol. 76: 1367–1395. 10.1152/jn.1996.76.3.1367 8890259

[37] Ghazanfar AA, Nicolelis MA (1997) Nonlinear processing of tactile information in the thalamocortical loop. J Neurophysiol. 78: 506–510. 10.1152/jn.1997.78.1.506 9242297

[38] Crick F (1984) Function of the thalamic reticular complex: the searchlight hypothesis. Proc Natl Acad Sci U S A. 81: 4586–4590. 10.1073/pnas.81.14.4586 PMC345636 6589612

[39] Steriade M, McCormick DA, Sejnowski TJ (1993) Thalamocortical oscillations in the sleeping and aroused brain. Science. 262: 679–685. 10.1126/science.8235588 8235588

[40] McCormick DA, Bal T (1997) Sleep and arousal: thalamocortical mechanisms. Annu Rev Neurosci. 20: 185–215. 10.1146/annurev.neuro.20.1.185 9056712

[41] Sherman SM (2001) Tonic and burst firing: dual modes of thalamocortical relay. Trends Neurosci. 24: 122–126. 10.1016/s0166-2236(00)01714-8 11164943

[42] Steriade M (2003) The corticothalamic system in sleep. Front Biosci. 8: 878–899. 10.2741/1043 12700074

[43] Huguenard JR (1999) Neuronal circuitry of thalamocortical epilepsy and mechanisms of antiabsence drug action. Adv Neurol. 79: 991–999. 10514881

[44] Shin HS (2006) T-type Ca2+ channels and absence epilepsy. Cell Calcium. 40: 191–196. 10.1016/j.ceca.2006.04.023 16777220

[45] Zaman T, Lee K, Park C, Paydar A, Choi JH, et al (2011) Cav2.3 channels are critical for oscillatory burst discharges in the reticular thalamus and absence epilepsy. Neuron. 70: 95–108. 10.1016/j.neuron.2011.02.042 21482359

[46] Destexhe A, Neubig M, Ulrich D, Huguenard J (1998) Dendritic low-threshold calcium currents in thalamic relay cells. J Neurosci. 18: 3574–3588. 10.1523/JNEUROSCI.18-10-03574.1998 PMC6793130 9570789

[47] Williams SR, Toth TI, Turner JP, Hughes SW, Crunelli V (1997) The ‘window’ component of the low threshold Ca2+ current produces input signal amplification and bistability in cat and rat thalamocortical neurones. J Physiol. 505: 689–705. 10.1111/j.1469-7793.1997.689ba.x PMC1160046 9457646

[48] Zhan XJ, Cox CL, Rinzel J, Sherman SM (1999) Current clamp and modeling studies of low-threshold calcium spikes in cells of the cat’s lateral geniculate nucleus. J Neurophysiol. 81: 2360–2373. 10.1152/jn.1999.81.5.2360 10322072

[49] Touboul J, Brette R (2009) Spiking Dynamics of Bidimensional Integrate-and-Fire Neurons. SIAM J. Appl. Dyn. Syst. 8: 1462–1506.

[50] Hirsh M, Pugh C, Shub M (1977) Invariant Manifolds. Lecture Notes in Mathematics. Springer-Verlag, Berlin, Germany.

[51] Morris C, Lecar H (1981) Voltage oscillations in the barnacle giant muscle fiber. Biophys J. 35: 193–213. 10.1016/S0006-3495(81)84782-0 PMC1327511 7260316

